# Supplementary material for: A paralog of Pcc1 is the fifth core subunit of the KEOPS tRNA-modifying complex in Archaea
Source: Nat Commun. 2023 Feb 1;14:526. doi: 10.1038/s41467-023-36210-y (PMC9889334; doi:10.1038/s41467-023-36210-y)
Supplement: Supplementary file 1 — Supplementary information [file 41467_2023_36210_MOESM1_ESM.pdf]

Supplementary Information File for

A paralog of Pcc1 is the fifth core subunit of the KEOPS tRNA-modifying complex in Archaea

Marie-Claire Daugeron[1,$], Sophia Missoury[1,$,§], Violette Da Cunha[1,£], Noureddine Lazar[1], Bruno Collinet[1,2] Herman van Tilbeurgh[1,#] & Tamara Basta[1,#].

[1] Université Paris-Saclay, CEA, CNRS, Institute for Integrative Biology of the Cell (I2BC), 91198, Gif-sur-Yvette, France

[2] Institut de Minéralogie de Physique des Matériaux et de Cosmochimie (IMPMC), Sorbonne-Université, UMR7590 CNRS, MNHN, Paris, France.

[#]Corresponding authors:

tamara.basta@i2bc.paris-saclay.fr

herman.van-tilbeurgh@i2bc.paris-saclay.fr

[$]These authors contributed equally

[£]Present address: Génomique Métabolique, Genoscope, Institut François Jacob, CEA, CNRS, Univ Evry, Université Paris-Saclay, 91057 Evry, France

[§]Present address: Department of structural biology and chemistry, Institut Pasteur, Paris, France.

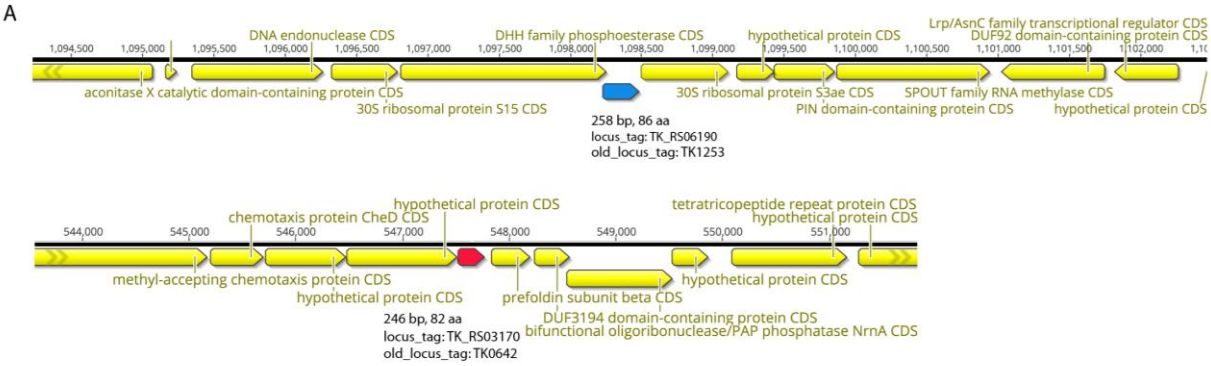

B

```
# Length: 108
# Identity:      32/108 (29.6%)
# Similarity:    49/108 (45.4%)
# Gaps:          49/108 (45.4%)
# Score: 133

PF2011  1 M--KAKRVQAKIEI-EFPSEDVAKV--V-YEAV-L--YEHLSVP--YRRS     39
          |  || ||:  | : .: .:| ||.  :  ||: :   |  |:|  .::|
TK1253  1 MEVKA-RVE--I-VWHY-GDD-AKARAIA-EAIQVDN-E--SMPEELKKS     40

PF2011 40 -EID-FK-LEG----K-KI---ILD--IKATDSSALRGTV-NSYLRWIKA     75
           .:.  : ::|    | |.   | |  |||.|.  :   |  | :: | |
TK1253 41 LNVQT-RWVDGDVITKVKYSGEI-DTLIKALDD--I---VF-S-VK-I-A     79

PF2011 76 AID-VIEV     82
          . | | ||
TK1253 80 E-DSV-EV     85
```

```
# Length: 92
# Identity:      51/92 (55.4%)
# Similarity:    69/92 (75.0%)
# Gaps:          20/92 (21.7%)
# Score: 266

PF2011  1 M-KAKRVQA-KIEIEFPSEDVAKVVYEAV-LYEH-LSVPYRRSE-IDF-K     44
          | |:. ::|  ||:|||:|:||:||||:| : ||  :||||||::  :|
TK0642  1 MMKSP-IEAV-IELEFPNEEVARVVYESVRI-EHD-TVPYRRTKG-EFV-     44

PF2011 45 LEGKKI-ILDIK--ATDSSALRGTVNSYLRWIKAAID-VIEV     82
          |:|:||  |  |  |.|:||||||:||||||||.|:|  |||
TK0642 45 LDGRKIR-L--KFIAEDNSALRGTLNSYLRWIKVAMDS-IEV     82
```

Supplementary figure 1. Identification of Pcc1 and Pcc1-like genes in *Thermococcus kodakarensis* KOD1.

A) Genomic context for TK1253 and TK0642 genes. Genome coordinates are indicated above the black line.

B) Pairwise alignment of Pcc1 protein sequence from *Pyrococcus furiosus* (PF2011) and TK1253 (left) or TK0642 (right).

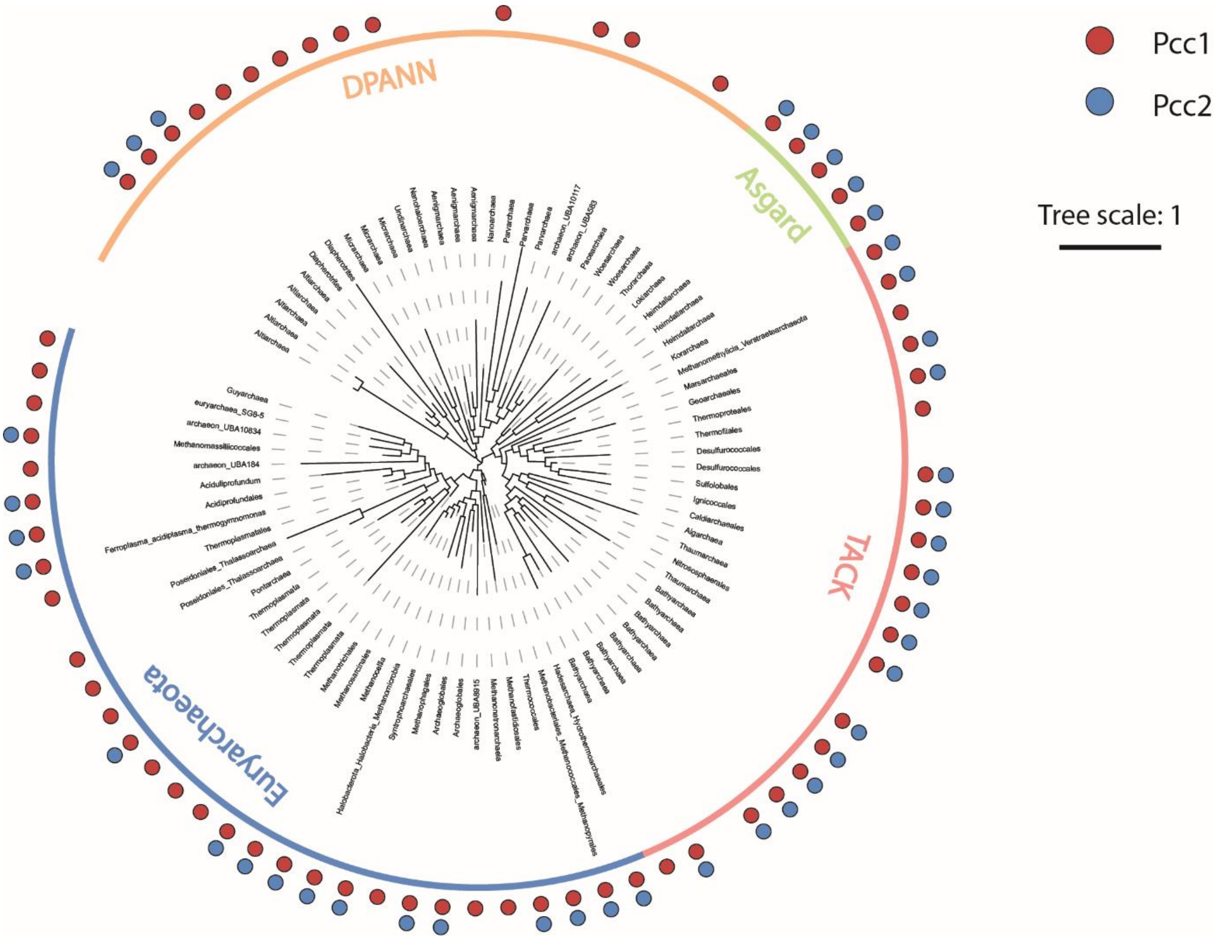

Supplementary figure 2. Distribution of Pcc1 and Pcc2 proteins in archaea

Distribution of Pcc1 (red circles) and Pcc2 (blue circles) mapped onto archaeal phylogenetic tree. The Pcc1 and Pcc2 sequences were detected using HMM searches. The leaves correspond to the class taxonomic level and they are further classified into four superphyla indicated by coloured lines. Tree scale indicates the correspondence between branch length and the amount of evolution (expressed as the number of substitutions per position in the alignment).

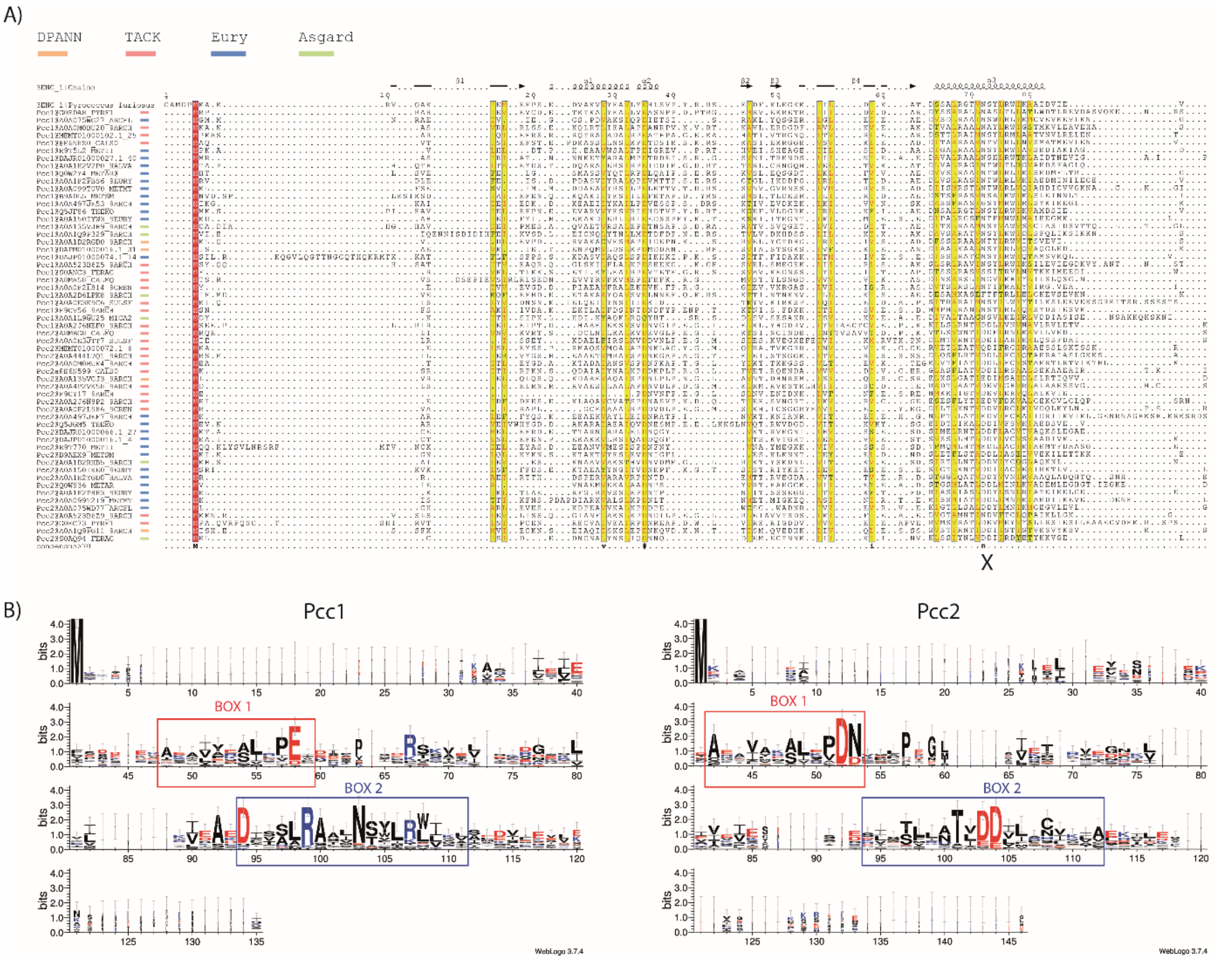

Supplementary figure 3. Sequence conservation of Pcc1 and Pcc2 proteins

A) An alignment of representative Pcc1 and Pcc2 sequences covering 27 archaeal phyla and the four superphyla. The names and the color code of the four superphyla are indicated above the alignment. Conserved residues are boxed in red, similar residues are boxed in yellow. The bottom line corresponds to the consensus sequence. The alignment was rendered with ESPript 3.0 using the crystal structure of Pcc1 from *Pyrococcus furiosus* as template (PDB 3ENC). The secondary structure elements are indicated above the alignment. X sign indicates the position, within the α3 helix, of the conserved residues N^{72}, S/T^{73} or D/E^{72}, D/E^{73} in Pcc1 and Pcc2 sequences, respectively.

B) Sequence logo calculated from the aforementioned alignment of Pcc1 or Pcc2 sequences. The logo was rendered using WebLogo 3 web application. Positively charged residues are colored in blue, negatively charged residues are colored in red. Of note, the logos are very similar to those obtained from the full set of identified Pcc1 and Pcc2 sequences (see materials and methods).

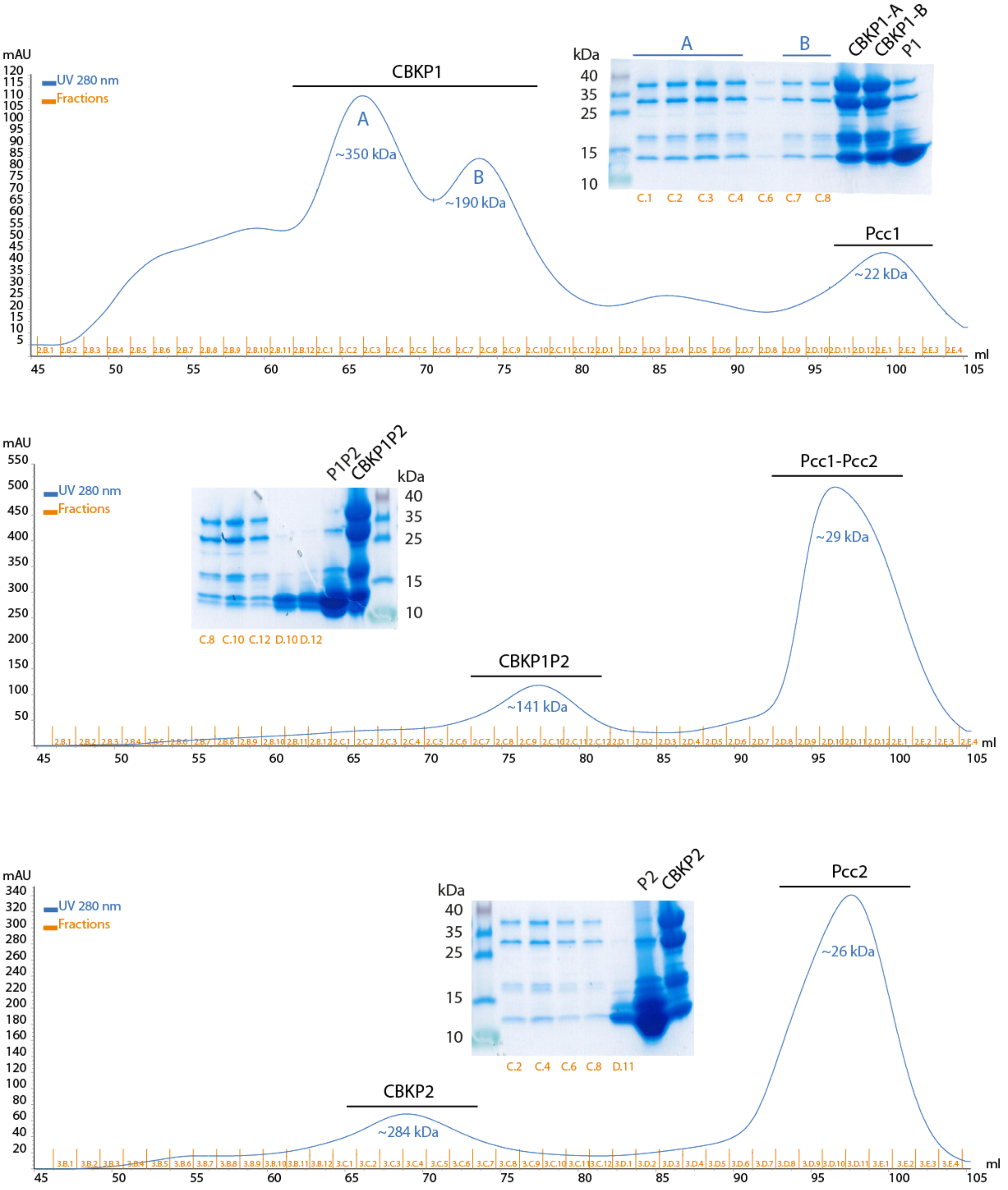

Supplementary figure 4. Purification of PaKEOPS complexes.

The separation of the proteins was done using HiLoadR 16/600 SuperdexR 200pg column in a buffer containing 500 mM of NaCl. For each complex the elution profile and the corresponding SDS-PAGE analysis is shown. The purification experiments were repeated at least four times with reproducible results.

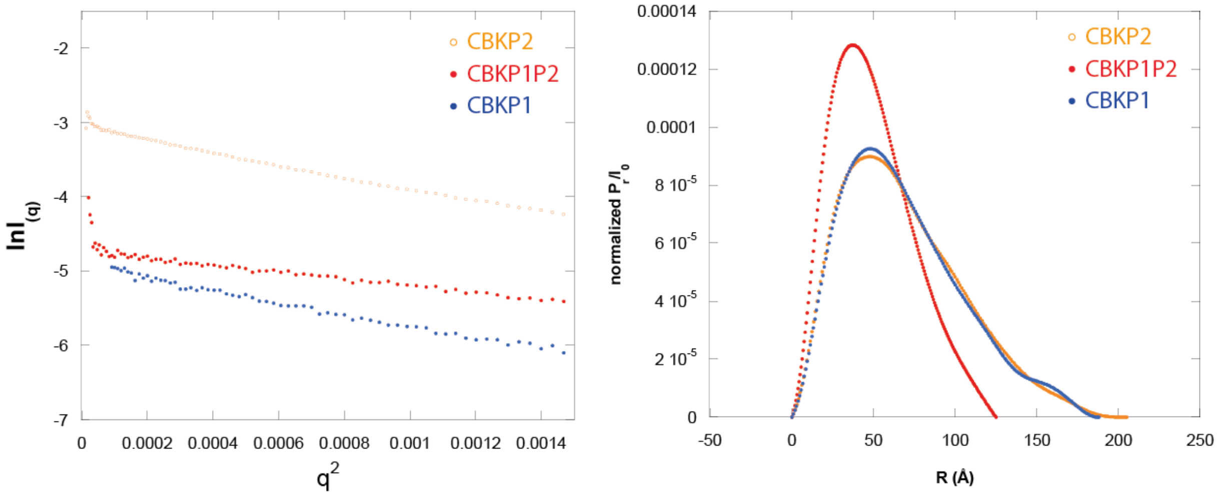

Supplementary figure 5. Analysis of small angle scattering data.

Guinier plot of the collected scattering data (left) and distance distribution (right) for CBKP2 (orange), CBKP1P2 (red) and CBKP1 (blue) complexes. The normalized distance distribution was calculated by PrimusQT implemented in Atsas software.

A

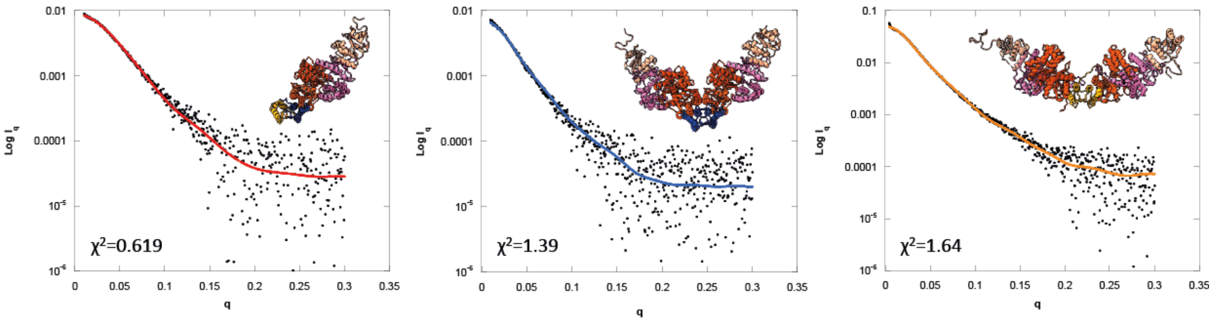

B

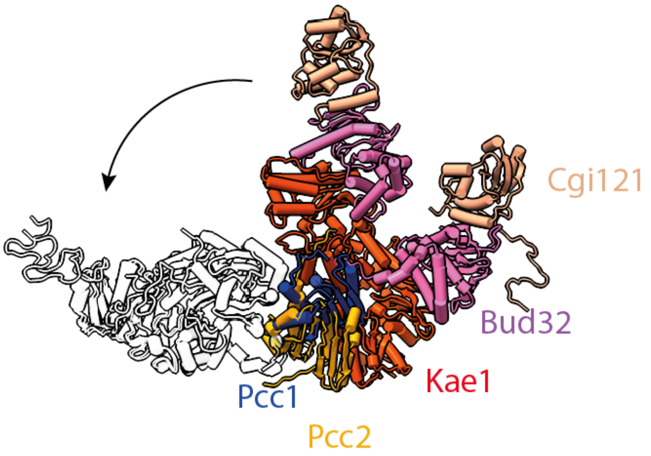

Supplementary figure 6. SAXS data and structural modelling of KEOPS complexes

A) SAXS scattering curves of the CBKP1P2 (left panel), CBKP1 (middle panel) and CBKP2 (right panel) complexes. The red line is the calculated scattering curve for the model represented in the top right corner of each plot. The reduced chi-square (χ^2) test compares the discrepancies between the theoretical and experimental curves with the expected errors.

B) Superposition of CBKP1 and CBKP2 model structures. The alignment of the two models over one heterotetramer shows that the second one is strongly deflected in the CBKP2 complex. The relative interaction of Pcc1 in blue with Kae1 in red and Pcc2 in orange with Kae1 is slightly different that applicate a huge deflection at the end of the octamere.

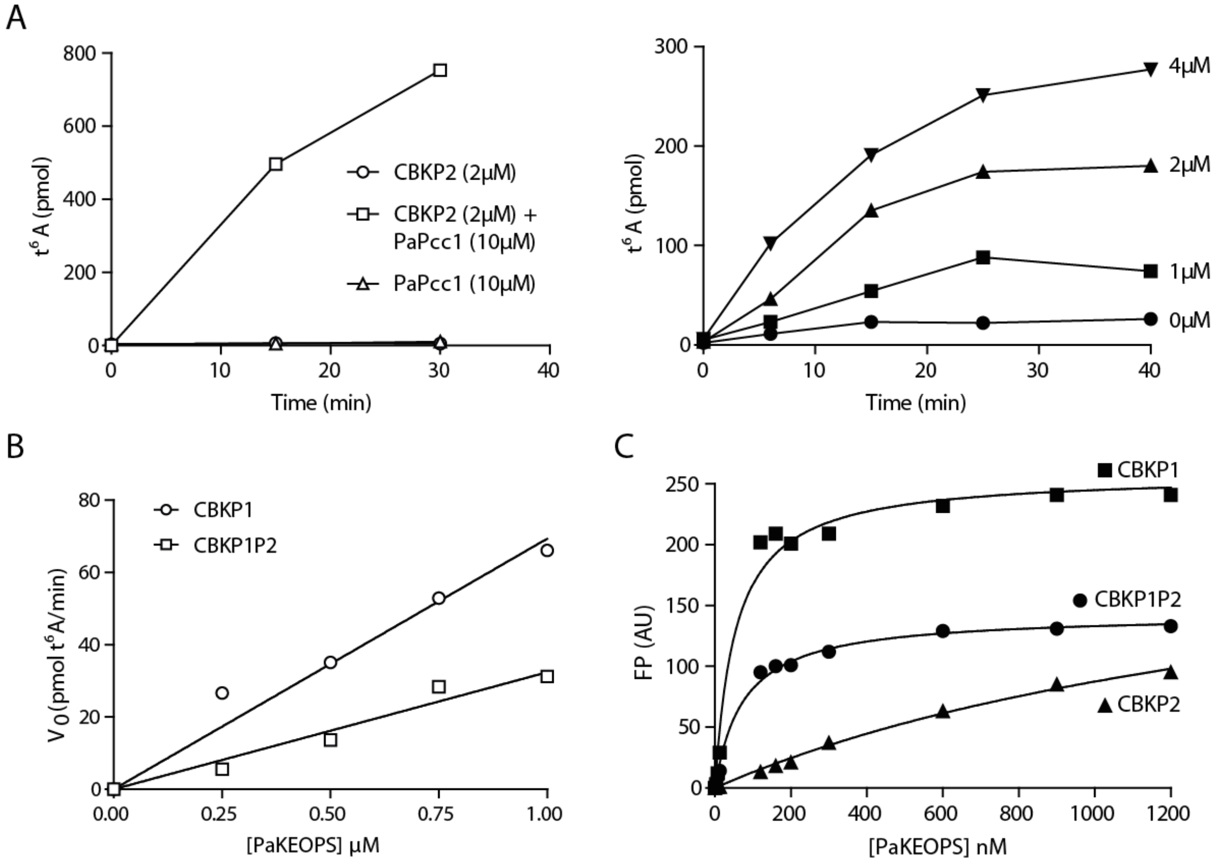

Supplementary figure 7. Activity and tRNA binding of PaKEOPS complexes.

A) The activity of CBKP2 can be restored by addition of PaPcc1. The t^6A formation was measured under standard conditions (see material and methods). The activity of CBKP2 (2µM final concentration) was restored by addition of PaPcc1 at 5-fold molar excess (left panel). PaPcc1 alone had no activity. The activity of CBKP2could be partially restored at equimolar (2µM) concentration of PaPcc1 (right panel).

B) Reaction velocities at different PaKEOPS complex concentrations. Maximal reaction velocity of t^6A synthesis was measured under steady state conditions using varying concentrations of CBKP1 or CBKP1P2 complexes. Data points were fitted with linear regression the slope of which is a measure of catalytic efficiency per complex. The slope is 2-fold higher for CBKP1complex.

C) tRNA binding of PaKEOPS complexes. The fluorescence polarisation signal was measured for complexes containing 10 nM of tRNA substrate and increasing concentration of CBKP1, CBKP1P2 and CBKP2 complexes. The data points were fitted to one-site specific binding equation to calculate the equilibrium dissociation constant. Source data are provided as a Source Data file.

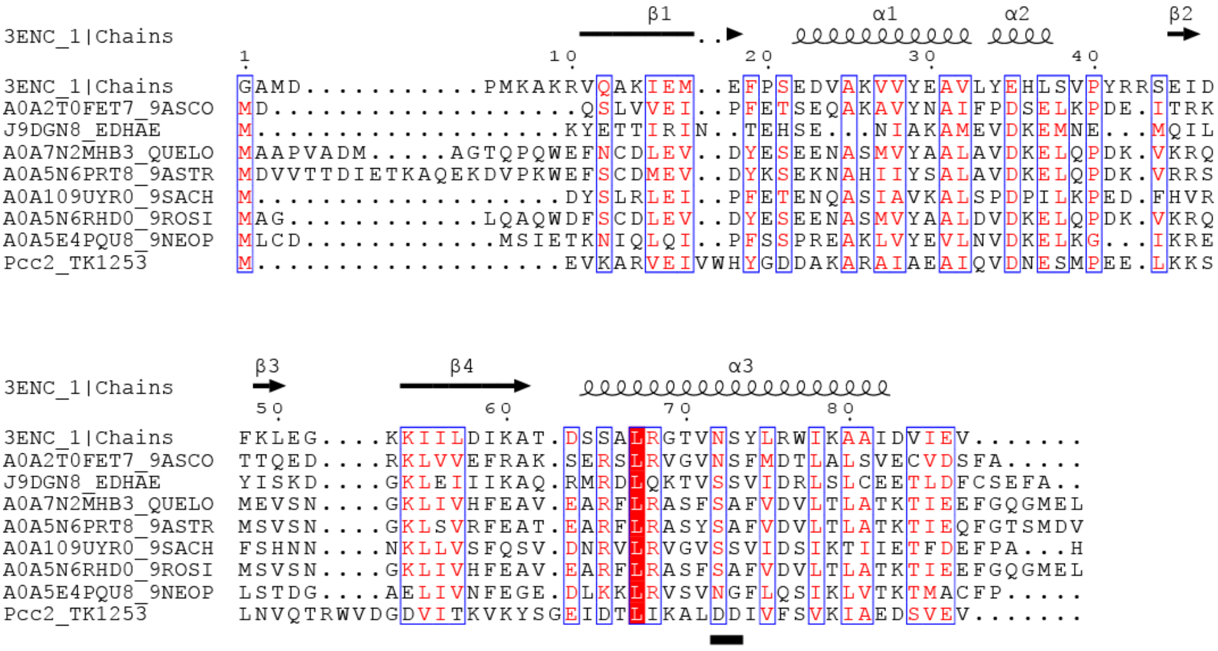

Supplementary figure 8. HMM-guided search for Pcc2 orthologs in Eukaryotes.

The alignment of 27 representative Pcc2 sequences was used to produce HMM profile which was subsequently used to search for Pcc2 orthologs in UniProtUK database with hmmsearch (version 2.41.2). The search was restricted to eukaryotic sequences. The Pcc1 of *P. furiosus* (PDB 3ENC) and Pcc2 of *T. kodakarensis* (TK1253) were used as reference sequences. The discriminating motif between Pcc1 and Pcc2 proteins is underlined. Residues with similar physico-chemical properties are indicated in red, conserved residues are boxed in red. The positions with global similarity score higher than the 0.7 threshold are framed in blue.

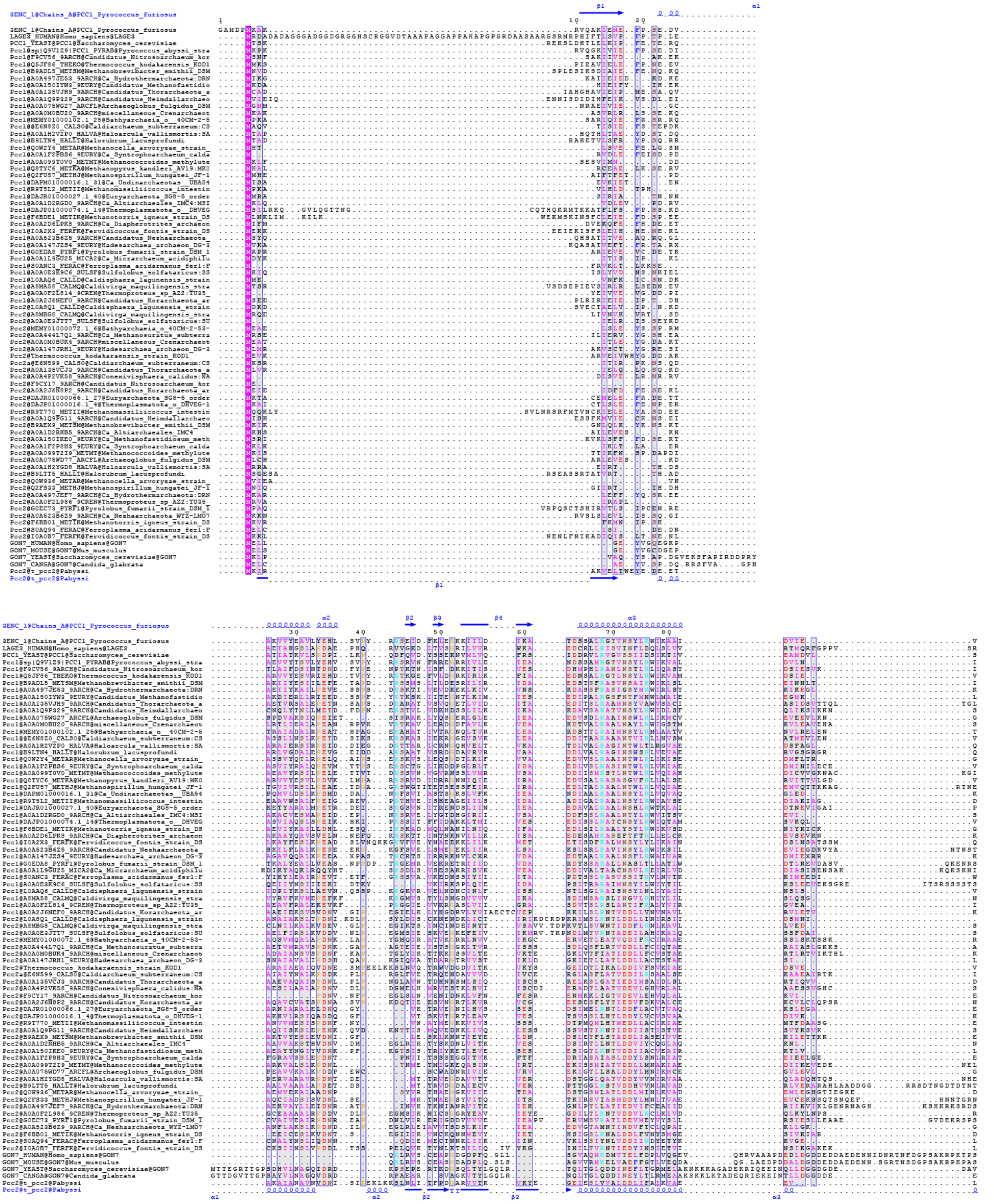

Supplementary figure 9. Alignment of archaeal Pcc1, Pcc2 and eukaryotic Gon7 and C14 sequences.

Sequences were aligned using MAFFT and rendered using ESPpript server. Crystal structures of PfPcc1 (3ENC) and Pcc2 (7A66) were used as reference for structure-guided alignment. Distribution of secondary structure elements (alpha helices and beta sheets) is indicated in the top and bottom line. Blue boxes signify that the positions in the alignment have similarity score

of more that 0.7 (with 1 being maximum). The residues are colored according to their physico-chemical properties.

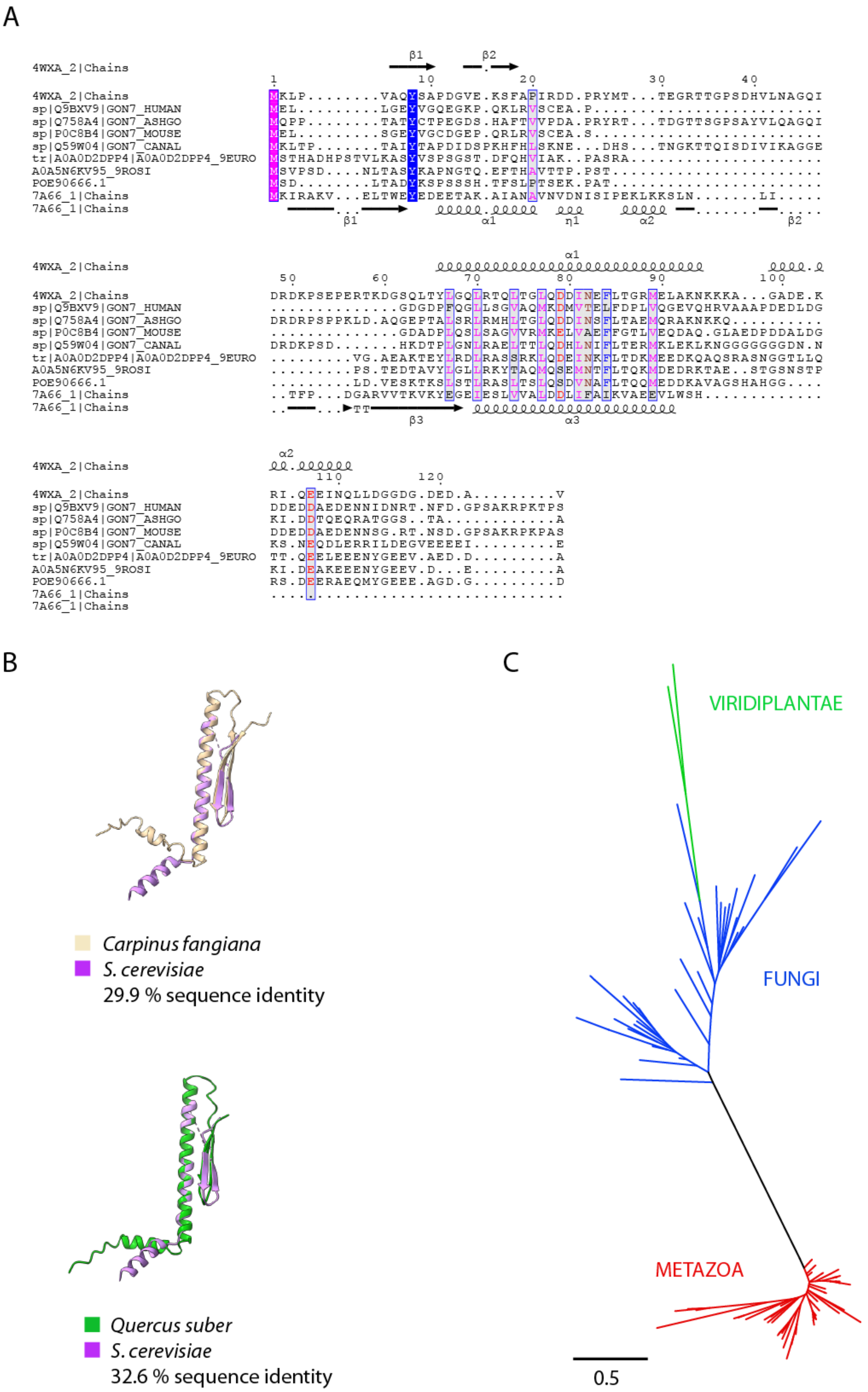

A
4WXA_2|Chains
sp|Q9BXV9|GON7_HUMAN
sp|Q758A4|GON7_ASHGO
sp|P0C8B4|GON7_MOUSE
sp|Q59W04|GON7_CANAL
tr|A0A0D2DPP4|A0A0D2DPP4_9EURO
A0A5N6KV95_9ROSI
POE90666.1
7A66_1|Chains
B
Carpinus fangiana
S. cerevisiae
29.9 % sequence identity
Quercus suber
S. cerevisiae
32.6 % sequence identity
C
VIRIDIPLANTAE
FUNGI
METAZOA
0.5

Supplementary figure 10. Sequence analysis of Gon7 proteins from plants.

A) Sequence alignment of representative Gon7 (*Ashbya gossypii*, *Candida albicans*, *Exophiala oligosperma*) and C14 (*Homo sapiens*, *Mus musculus*) sequences including the two orthologs from plants (*Carpinus fangiana* and *Quercus suber*). Secondary structure distribution of Gon7 from *S. cerevisiae* (PDB 4WXA) and PaPcc2 (PDB 7A66) is shown on the top and below the alignment, respectively. Colors indicate physico-chemical properties of amino acids and positions with similarity score > 0.7 are boxed.

B) Model structures of Gon7 from *Carpinus fangiana* and *Quercus suber*. Structures were predicted using AlphaFold2 and visualized with ChimeraX1.3.

C) Phylogenetic tree of Gon7 and C14 orthologs. The sequences from Fungi (ENOG503P560, 32 sequences) and Metazoa (ENOG503BUEY, 60 sequences) were retrieved from Eggnog database and aligned with T-coffee. The maximum likelihood tree was inferred using IQ-tree and visualized with iTOL. The scale bar represents the amount of sequence substitution per position in alignment.

TAATACGACTCACTATAGGGAGATCTACCGGCCCCTGATGAGTCCGTGAGGACGAAACGCTA
CCCGGTAGCGTCGGGCCGGTAGCTCAGCCTGGTCAGAGCACCGGGCTTTTAACCCGGTGGTC
GCGGGTTCAAATCCCGCCCGGCCCGCCAGCGCCCGAACTACCGGTACCGGTAGTTGACGAGG
ATGGAGGTTATCGAATTTTCGGCGGATGCCTCCCGGCTGAGTGTGCAGATCACAGCCGTAAG
GATTTCTTCAAACCAAGGGGGTGACTCCTTGAACAAAGAGAAATCACATGATCTTCCTTTTT
TCTAGACGTACACCATCAGGGTACGTTTTTCAGACACCATCAGGGTCTGACGTCTTC

Supplementary figure 11. Sequence of the tRNA expression cassette.

The cassette consists from 5’ to 3’ end of: hammerhead ribozyme carrying T7 promotor, tRNA gene, glmS ribozyme and a double MS2 tag. tRNA gene encoding tRNA^{Lys} (UUU) from *P. abyssi* is underlined.

	PaPcc2	PaPcc1-PaPcc2
PDB code	7A66	7A67
Number of molecules in asymmetric unit	3	2
Space group<br><br>Cell parameters (Å, °)	C 1 2 1<br><br>a=76.80 b=81.31 c=66.48<br>α=90.00 β=121.59 γ=90.00	P 32 2 1<br><br>a=b=69.90 c=92.75<br>α= β =90.00 γ =120.00
Resolution (Å)	50-1.84<br>(1.96-1.84)	50.00-3.18<br>(3.36-3.18)
X-ray wavelength	0.983988	0.980066
No. of total reflections	205991<br>(32315)	87363<br>(11857)
No. of unique reflections	29812<br>(4675)	4659<br>(678)
Completeness (%)	99.5<br>(96.9)	98.4<br>(91)
I/σ	18.24<br>(1.08)	10.54<br>(1.54)
CC1/2	99.9<br>(65.5)	99.7<br>(85.9)
Rcryst	0.204 (0.239)	0.185 (0.235)
Rfree	0.229 (0.3270)	0.250 (0.488)
RMSD bond lengths (Å)	0.010	0.010
RMDS bond angles (°)	1.12	1.25
Average B ($Å^2$)	45.0	60.0
Clash score<br>MolProbity score	2.07<br>0.98	10.5<br>3.09
Ramachandran plot (%)<br>Favored<br>Allowed<br>Outliers	 98.37<br>1.63<br>0.00	 89.38<br>9.37<br>1.25

Supplementary table 1. X-ray data collection and structure refinement statistics. Values for the highest resolution shell are in parentheses. $CC_{1/2}$ = percentage of correlation between intensities from random half-dataset. [a]Calculated with MolProbity (MolProbity score combines the clash score, rotamer, and Ramachandran evaluations into a single score, normalized to be on the same scale as X-ray resolution).

	CBKP1P2	CBKP1	CBKP2
Data collection parameters			
Instrument	SWING (SOLEIL)		
Detector	CCD-based AVIEX		
Beam geometry	0.8 mm x 0.15 mm		
Wavelength [Å]	1.0		
q-range [$Å^{-1}$]	0.007 < q < 0.50		
Absolute scaling	Comparison with scattering from pure H_2O		
Exposure time [s]	2		
Temperature [K]	283		
SEC-SAXS column	Biosec 3 Agilent		
Loading concentration [mg/mL]	2.55	0.72	3
Injection volume [µL]	50		
Flow rate [$mL.min^{-1}$]	0.3		
Structural parameters			
I(0) Guinier [cm^{-1}]	$8.75\ 10^{-3}$	$7.46\ 10^{-3} \pm 8.44\ 10^{-5}$	$4.77\ \ 10^{-2} \pm 1.13\ 10^{-4}$
R_g Guinier [Å]	36.9	52.5± 0.873	52.4 ± 0.19
qRg-range	0.45-1.3	0.479-1.29	0.47-1.27
I(0) p(r) [cm^{-1}]	$8.90\ 10^{-3}$	7.54 10-3 ± 0.90-4	$4.83\ 10^{-2} \pm 1.22\ 10^{-5}$
R_g p(r) [Å]	38.87 ± 0.23	54.42 ± 0.88	54.89 ± 0.22
q-range [$Å^{-1}$]	0.0091-0.2639	0.091 – 0.197	0.0078 – 0.1856
D_{Max} [Å]	125	188	205
Molecular mass determination			
Mol . L -1$MM_{sequence}$ [kDa] [b]	101.457	91.146	90.871
$MM_{bayesian}$ [kDa] [c]	~94.225	~185.775	~180.5
Credibility interval	85.6-100.3	162.65-194.95	151.45-194.95

Supplementary table 2. SAXS results and data collection parameters.
