# Supplementary material for: A paralog of Pcc1 is the fifth core subunit of the KEOPS tRNA-modifying complex in Archaea
Source: Nat Commun. 2023 Feb 1;14:526. doi: 10.1038/s41467-023-36210-y (PMC9889334; doi:10.1038/s41467-023-36210-y)
Supplement: Supplementary file 3 — Description of Additional Supplementary Files [file 41467_2023_36210_MOESM3_ESM.pdf]

Description of Additional Supplementary Files:

Supplementary Dataset 1: Sequence homology search for Pcc1 and Pcc2 orthologs. The table contains raw data and the summary of the raw data from the Jackhmmr search.

Source data: Enzymatic activity and tRNA binding of archaeal KEOPS complexes. The file contains raw data obtained for measurements of t6 A formation and tRNA binding using purified KEOPS complexes.
