# Supplementary material for: A paralog of Pcc1 is the fifth core subunit of the KEOPS tRNA-modifying complex in Archaea
Source: Nat Commun. 2023 Feb 1;14:526. doi: 10.1038/s41467-023-36210-y (PMC9889334; doi:10.1038/s41467-023-36210-y)
Supplement: Supplementary file 5 — Reporting Summary [file 41467_2023_36210_MOESM5_ESM.pdf]

nature portfolio

Corresponding author(s): Tamara Basta, Herman van Tilbeurgh

Last updated by author(s): Dec 24, 2022

Reporting Summary

Nature Portfolio wishes to improve the reproducibility of the work that we publish. This form provides structure for consistency and transparency in reporting. For further information on Nature Portfolio policies, see our Editorial Policies and the Editorial Policy Checklist.

Statistics

For all statistical analyses, confirm that the following items are present in the figure legend, table legend, main text, or Methods section.

n/a	Confirmed	
☐	☒	The exact sample size (*n*) for each experimental group/condition, given as a discrete number and unit of measurement
☒	☐	A statement on whether measurements were taken from distinct samples or whether the same sample was measured repeatedly
☐	☒	The statistical test(s) used AND whether they are one- or two-sided<br>*Only common tests should be described solely by name; describe more complex techniques in the Methods section.*
☒	☐	A description of all covariates tested
☒	☐	A description of any assumptions or corrections, such as tests of normality and adjustment for multiple comparisons
☒	☐	A full description of the statistical parameters including central tendency (e.g. means) or other basic estimates (e.g. regression coefficient) AND variation (e.g. standard deviation) or associated estimates of uncertainty (e.g. confidence intervals)
☒	☐	For null hypothesis testing, the test statistic (e.g. F, t, r) with confidence intervals, effect sizes, degrees of freedom and P value noted<br>*Give P values as exact values whenever suitable.*
☒	☐	For Bayesian analysis, information on the choice of priors and Markov chain Monte Carlo settings
☒	☐	For hierarchical and complex designs, identification of the appropriate level for tests and full reporting of outcomes
☒	☐	Estimates of effect sizes (e.g. Cohen's *d*, Pearson's *r*), indicating how they were calculated

Our web collection on statistics for biologists contains articles on many of the points above.

Software and code

Policy information about availability of computer code

Data collection	Jackhmmr program (https://www.ebi.ac.uk/Tools/hmmer/search/jackhmmer), EggNOG database 5.0.0
Data analysis	MAFFT V7, IQ-TREE v1.7, CCP4 suite, PHASER, MODELLER, COOT, BUSTER, FoxTrot50, PrimusQT, Dadimodo, FoxsDock, CRYSOL, ESPRIPT web-server (https://espript.ibcp.fr/ESPript/ESPript/), Pymol v.2, PDBeFold web server (https://www.ebi.ac.uk/msd-srv/ssm/), PDBePISA web server (https://www.ebi.ac.uk/pdbe/pisa/)

For manuscripts utilizing custom algorithms or software that are central to the research but not yet described in published literature, software must be made available to editors and reviewers. We strongly encourage code deposition in a community repository (e.g. GitHub). See the Nature Portfolio guidelines for submitting code & software for further information.

Data

Policy information about availability of data

All manuscripts must include a data availability statement. This statement should provide the following information, where applicable:

- Accession codes, unique identifiers, or web links for publicly available datasets
- A description of any restrictions on data availability
- For clinical datasets or third party data, please ensure that the statement adheres to our policy

The manually curated set of Pcc1 and Pcc2 sequences used in this study are available at https://doi.org/10.6084/m9.figshare.21640394.v1. The raw data from HMM-based searches are available as Supplementary Data 1. The crystal structures of Pcc1 and Pcc2 proteins were deposited to the PDB database, accession numbers 7A66 [http://doi.org/10.2210/pdb7A66/pdb] and 7A67 [http://doi.org/10.2210/pdb7A67/pdb]. Source data are provided with this paper.

Human research participants

Policy information about studies involving human research participants and Sex and Gender in Research.

Reporting on sex and gender	not applicable
Population characteristics	not applicable
Recruitment	not applicable
Ethics oversight	not applicable

Note that full information on the approval of the study protocol must also be provided in the manuscript.

Field-specific reporting

Please select the one below that is the best fit for your research. If you are not sure, read the appropriate sections before making your selection.

[x] Life sciences [ ] Behavioural & social sciences [ ] Ecological, evolutionary & environmental sciences

For a reference copy of the document with all sections, see nature.com/documents/nr-reporting-summary-flat.pdf

Life sciences study design

All studies must disclose on these points even when the disclosure is negative.

Sample size	Enzymatic assays were performed in triplicates or quadruplicates. The data were presented as mean values with error bars indicating standard deviation from the mean.
Data exclusions	No data was excluded.
Replication	Analytical experiments were conducted at least in triplicates and were reproducible. Crystallisation assays and the crystal structure resolution using Soleil synchrontron were conducted once.
Randomization	There are no experimental groups in this study.
Blinding	There are no groups in our study.

Reporting for specific materials, systems and methods

We require information from authors about some types of materials, experimental systems and methods used in many studies. Here, indicate whether each material, system or method listed is relevant to your study. If you are not sure if a list item applies to your research, read the appropriate section before selecting a response.

Materials & experimental systems

n/a	Involved in the study
☒	☐ Antibodies
☒	☐ Eukaryotic cell lines
☒	☐ Palaeontology and archaeology
☒	☐ Animals and other organisms
☒	☐ Clinical data
☒	☐ Dual use research of concern

Methods

n/a	Involved in the study
☒	☐ ChIP-seq
☒	☐ Flow cytometry
☒	☐ MRI-based neuroimaging
